# Timing of recombinant human granulocyte colony-stimulating factor administration on neutropenia induced by cyclophosphamide in normal mice.

**DOI:** 10.1038/bjc.1998.146

**Published:** 1998-03

**Authors:** M. Misaki, Y. Ueyama, G. Tsukamoto, T. Matsumura

**Affiliations:** Department of Oral and Maxillofacial Surgery II, Okayama University Dental School, Japan.

## Abstract

The effects of altering the timing of recombinant human granulocyte colony-stimulating factor (rhG-CSF) administration on neutropenia induced by cyclophosphamide (CPA) were studied experimentally in a mouse model. Experimental mice were divided into three groups: (a) treatment with rhG-CSF after CPA administration (post-treatment group); (b) treatment with rhG-CSF both before and after CPA administration (pre- and post-treatment group); and (c) treatment with saline after CPA administration (control group). The results were as follows. Mice receiving rhG-CSF on the 2 days preceding CPA treatment, in which progenitor cell counts outside the S-phase when CPA was administered were the lowest of all the groups, showed accelerated neutrophil recovery but decreased neutrophil nadirs compared with the control group despite rhG-CSF treatment. The pre- and post-treatment group, consisting of mice who received rhG-CSF treatment on days -4 and -3 before CPA treatment, and in which progenitor cell counts when CPA was administered were increased to greater levels than in the other groups, showed remarkably accelerated neutrophil recovery and the greatest increase in the neutrophil nadirs of all the groups. These results suggested that the kinetics of progenitor cell populations when chemotherapeutic agents were administered seemed to play an important role in neutropenia after chemotherapy, and that not only peripheral neutrophil cell and total progenitor cell counts but also progenitor cell kinetics should be taken into consideration when administering rhG-CSF treatment against the effects of chemotherapy.


British Journal of Cancer (1998) 77(6), 884–889
© 1998 Cancer Research Campaign

### Timing of recombinant human granulocyte colony-stimulating factor administration on neutropenia induced by cyclophosphamide in normal mice

**M Misaki, Y Ueyama, G Tsukamoto and T Matsumura**

Department of Oral and Maxillofacial Surgery II, Okayama University Dental School, Japan

**Summary** The effects of altering the timing of recombinant human granulocyte colony-stimulating factor (rhG-CSF) administration on neutropenia induced by cyclophosphamide (CPA) were studied experimentally in a mouse model. Experimental mice were divided into three groups: (a) treatment with rhG-CSF after CPA administration (post-treatment group); (b) treatment with rhG-CSF both before and after CPA administration (pre- and post-treatment group); and (c) treatment with saline after CPA administration (control group). The results were as follows. Mice receiving rhG-CSF on the 2 days preceding CPA treatment, in which progenitor cell counts outside the S-phase when CPA was administered were the lowest of all the groups, showed accelerated neutrophil recovery but decreased neutrophil nadirs compared with the control group despite rhG-CSF treatment. The pre- and post-treatment group, consisting of mice who received rhG-CSF treatment on days –4 and –3 before CPA treatment, and in which progenitor cell counts when CPA was administered were increased to greater levels than in the other groups, showed remarkably accelerated neutrophil recovery and the greatest increase in the neutrophil nadirs of all the groups. These results suggested that the kinetics of progenitor cell populations when chemotherapeutic agents were administered seemed to play an important role in neutropenia after chemotherapy, and that not only peripheral neutrophil cell and total progenitor cell counts but also progenitor cell kinetics should be taken into consideration when administering rhG-CSF treatment against the effects of chemotherapy.

**Keywords:** recombinant human granulocyte colony stimulating-factor; the timing of rhG-CSF administration; cyclophosphamide; chemotherapy

Neutropenia is a major factor contributing to infection and mortality in patients undergoing chemotherapy for cancer. It can limit drug dosages and the frequency of treatment and is one of the dose-limiting factors in anti-cancer chemotherapy. Recombinant human granulocyte colony-stimulating factor (rhG-CSF) promotes proliferation and differentiation of granulocyte colony-forming progenitor cells (progenitor cells) and induces an increase in neutrophils in peripheral blood (Okabe et al, 1990; Takaue et al, 1990; Tanaka et al, 1991). Recently, clinical studies on rhG-CSF administration after chemotherapy have reported that rhG-CSF induces accelerated recovery from neutropenia, as evidenced by leucocyte or neutrophil counts (Gabrilove et al, 1988; Crawford et al, 1991; Sarosy et al, 1992). However, changes in granulopoietic activity with alterations in the timing of rhG-CSF administration on neutropenia induced by chemotherapy still remains unclear. Furthermore, expansions in granulocyte progenitor cell populations, which play an important role in granulopoiesis, have not been investigated in detail. By investigating the expansion of progenitor cell populations, as well as neutrophil counts, we studied the effects of altering the timing of rhG-CSF administration on neutropenia induced by cyclophosphamide (CPA) in normal mice.

*Received 28 May 1997*
*Revised 5 August 1997*
*Accepted 20 August 1997*

*Correspondence to*: Y Ueyama, 2-5-1 Shikata-cho, Okayama 700, Japan

### MATERIALS AND METHODS

### Mice and rhG-CSF administration

Female C57BL mice, aged 8–10 weeks, received a single 220 mg $kg^{-1}$ i.p. dose of CPA (Shionogi Seiyaku, Tokyo, Japan). Experimental mice also received 5 mg per body per day rhG-CSF (Kyowa Hakko Kogyo, Tokyo, Japan) s.c. in a 0.2-ml volume on the following schedule: treatment with rhG-CSF after CPA administration (post-treatment group), treatment with rhG-CSF both before and after CPA administration (pre- and post-treatment group). After investigating the effects of 2 days of rhG-CSF pretreatment on progenitor cells, the pre- and post-treatment group was subdivided into another two groups according to the number of progenitor cells outside the DNA synthesis when CPA was administered. In group A, CPA was administered when progenitor cell counts, excluding cells in the S-phase, were reduced by rhG-CSF pretreatment that was given on days –1 and –2. In group B, CPA was administered when progenitor cell counts, excluding cells in the S-phase, peaked after rhG-CSF pretreatment that was given on days –3 and –4. Control mice received 0.2 ml of 0.9% sodium chloride on each of 4 days after CPA administration (Table 1).

### Peripheral blood neutrophils

Twenty-four hours after injection, blood samples were obtained by cardiac puncture and total leucocyte counts were determined. Differential leucocyte counts were analysed on Wright–Giemsa-stained blood smears and final neutrophil counts were determined.

### Colony-forming unit assay

Twenty-four hours after injection, bone marrow nucleated cells were obtained aseptically from the femur. To evaluate granulocyte colony formation, bone marrow cells were suspended at $1 \times 10^5$ cells per ml in 0.3% agar containing α-MEM, 20% fetal calf serum (FCS), and 40 ng per ml of rhG-CSF. Cells were then transferred to 35-mm culture dishes, and incubated at 37°C in 5% carbon-dioxide for at least 7 days. Colonies containing more than 40 cells were enumerated as progenitor cells and were expressed as the number of colonies per femur.

### [$^3$H]thymidine suicide assay

Bone marrow nucleated cells were suspended at a density of $2 \times 10^6$ cells per ml of α-MEM. Cell suspensions (1.0 ml) were incubated with or without [$^3$H]thymidine (spec. act. 925 GBq mmol$^{-1}$, Amersham, Little Chalfont, Buckinghamshire, UK) for 20 min. Subsequently, cells were washed with α-MEM containing 100 μg ml$^{-1}$ unlabelled thymidine, and then assayed for colony-forming ability. Cells exposed to high specific activity [$^3$H]thymidine during DNA synthesis undergo a loss of colony-forming ability because of the lethal effect of the decay of incorporated tritium. The percentage of progenitor cells during the DNA synthesis (suicide rate) was determined by a comparison of colony numbers after in vitro treatment with high specific activity [$^3$H]-thymidine, and treatment without [$^3$H]thymidine. Subsequently, the number of progenitor cells in the S-phase (S-phase progenitor cells) and the number not in the S-phase (progenitor cells outside the S-phase) was enumerated.

### Statistical analysis

The average number of neutrophils, total progenitor cells, S-phase progenitor cells and progenitor cells outside the S-phase were analysed by the Mann–Whitney test (Bruning et al, 1987).

### RESULTS

### Effects of 2 days of rhG-CSF administration on neutrophil counts and expansion of progenitor cell populations (Figure 1)

To evaluate the effects of short-term rhG-CSF administration as a pretreatment, we injected rhG-CSF for 2 consecutive days and evaluated peripheral neutrophil counts as well as the expansion of granulocyte progenitor cell populations. Immediately after rhG-CSF administration, neutrophil counts increased gradually and reached a peak on day 2, after which neutrophil counts returned to control values.

Progenitor cell counts peaked on day 1 after rhG-CSF administration. Subsequently, in contrast to neutrophil counts, progenitor cell counts returned to control values on day 2 and then showed a slight increase on day 4.

Suicide rates gradually increased in accordance with rhG-CSF administration and peaked (85%) on day 2. Remarkably, the suicide rate decreased to nadir (11%) on day 4 and the S-phase progenitor cells similarly decreased to nadir. In contrast, the number of progenitor cells not in the S-phase decreased on day 2, and then increased markedly on day 4.

### Effects of post-treatment with rhG-CSF on neutrophil counts and progenitor cell counts after CPA administration (Figure 2)

In control mice, neutrophil counts after CPA administration gradually decreased to nadir from day 3 to day 4. This decrease was followed by a rapid recovery on day 5. Conversely, progenitor cell counts dropped rapidly to nadir on day 1, and recovered by day 3. In the post-treatment group, although the neutrophil count nadirs were similar to that in the control group, neutrophil counts increased rapidly on day 4 and there was a shortening of the period of neutropenia. However, in the post-treatment group, progenitor cell counts did not rise above initial levels until day 4, and recovery was not promoted despite rhG-CSF administration.

### Effects of post-treatment with rhG-CSF on the suicide rate and S-phase progenitor cells after CPA administration (Figure 3)

The suicide rate in both groups, which was approximately 60% before CPA administration, dropped markedly to 0% on day 1 and S-phase progenitor cells disappeared completely after CPA administration. Subsequently, suicide rates rose to about 80% on day 2, and there was a rapid recovery above the starting level, which was sustained until day 4.

S-phase progenitor cells in both control group and post-treatment with rhG-CSF disappeared on the next day after CPA administration and progenitor cells outside the S-phase only existed on the day after CPA administration. In the control group, S-phase progenitor cell counts increased considerably from day 3 to day 4, whereas in post-treatment with rhG-CSF group they had only a tendency to increase slightly, and S-phase progenitor cell counts did not increase compared with the control group, despite rhG-CSF administration.

### Effect of pre- and post-treatment with rhG-CSF on the suicide rate and S-phase progenitor cell counts after CPA administration (Figure 4)

In both group A and group B, the suicide rate dropped to 0% on day 1 after CPA administration, then showed rapid recovery on day 2. All of the S-phase progenitor cells disappeared completely on day 1. In group A, despite rhG-CSF administration, S-phase progenitor cell counts decreased from day 2 to day 4 compared with the control group. However, in group B, recovery of S-phase progenitor cells was markedly promoted until populations peaked at day 4.

### Effects of pre- and post-treatment with rhG-CSF on neutrophil counts and progenitor cell counts.

In group A (Figure 6), neutrophil counts decreased from day 1 to day 3 compared with the control group, and the neutrophil count nadir decreased in all groups, although shortening of the period of neutropenia was achieved on day 4. Progenitor cell counts also decreased from day 1 to day 3 compared with the control group, and the nadir was further reduced in all groups.

In contrast, group B showed the highest neutrophil count nadir and the most accelerated neutrophil recovery of all groups from day 2 to day 4 (Figure 6). The progenitor cell count nadir increased and recovery was promoted in all groups.

© Cancer Research Campaign 1998 

**Figure 1** Different protocols of rhG-CSF administration against CPA

### DISCUSSION

In this study, we investigated the effects of altering the timing of rhG-CSF administration on CPA-induced neutropenia and examined the possibility that pretreatment with rhG-CSF could reduce or prevent CPA-induced neutropenia.

Based on known actions of rhG-CSF in vitro and in vivo (Okabe et al, 1990; Takaue et al, 1990; Tanaka et al, 1991), the administration of rhG-CSF would be expected to induce an increase in the number of both neutrophils and bone marrow progenitor cells. However, in our study, total bone marrow progenitor cells did not show a constant increase as neutrophil counts despite rhG-CSF administration. The granulopoietic effect of rhG-CSF is to stimulate maturation and mobilization of neutrophils from the bone marrow compartment, as well as to stimulate proliferation and differentiation of progenitor cells (Okabe et al, 1990). Thus, this discrepancy between neutrophil and progenitor cell counts during rhG-CSF administration was probably because rhG-CSF administration induced an increase in neutrophil cell counts at the expense of progenitor cells in the progenitor pool. The suicide rate increased as rhG-CSF continued and showed that most of the progenitor cells were synchronized in S-phase. Although total progenitor cell

**Figure 2** Effects of rhG-CSF administrations on neutrophil counts and expansions of progenitor cells. rhG-CSF was administered from day 0 and 24 h later on day 1. Each data point represents mean value and bars represent s.d. ($n = 6$)

© Cancer Research Campaign 1998

**Figure 3** Effects of post-treatment with rhG-CSF on neutrophil counts and progenitor cell counts. In post-treatment group, CPA was administered on day 0, and rhG-CSF was administered from days 1 to 4. Each data point represents the mean value and bars represent s.d. ($n$ = 6, $^{**}P$ < 0.01 vs control group) respectively. Open symbols, control; closed symbols, post-treatment group

**Figure 4** Effects of post-treatment with rhG-CSF on suicide rate and S-phase progenitor cell counts. In post-treatment group, CPA was administered on day 0, and rhG-CSF was administered from days 1 to 4. Each data point represents the mean value and bars represent s.d. ($n$ = 6, $^{**}P$ < 0.01 vs control group) respectively. Open symbols, control; closed symbols, post-treatment group

**Figure 5** Effects of pre- and post-treatment with rhG-CSF on suicide rate and S-phase progenitor cell counts. Each data point represents the mean and bars represent s.d. ($n$ = 6, $^{**}P$ < 0.05 vs control group) respectively. ○, control; ■, group A; ●, group B

counts were maintained 3 days after the cessation of rhG-CSF, the suicide rate decreased and S-phase progenitor cells decreased markedly to nadir. This unexpected decrease in the suicide rate and S-phase progenitor cell counts has not been previously demonstrated, and the reason for these effects remains unclear. Because this decrease in the suicide rate and S-phase progenitor cells was not accompanied by an increase in either the total progenitor cell count or the neutrophil count, this decrease is not likely to be attributable to either proliferation of progenitor cells or differentiation to neutrophils at the expense of S-phase progenitor cells, as promoted by rhG-CSF. Moreover, total progenitor cell counts were maintained despite a decrease in the S-phase fraction. Thus, it is conceivable that most of the progenitor cells, after synchronization in S-phase after rhG-CSF administration, moved from S-phase into another phase of the cell cycle simultaneously. These observations suggested that rhG-CSF administration affected not only the total number of progenitor cells but also the kinetics of progenitor cells even after the cessation of rhG-CSF administration. Moreover, the effects of rhG-CSF on neutrophil and progenitor cells in vivo were revealed to be quite different.

Regarding the effect of rhG-CSF administration before chemotherapy, Morstyn et al (1989) reported that pretreatment with rhG-CSF in addition to post-treatment did not increase the nadir and there was the potential risk of inducing neutrophil exhaustion with rhG-CSF pretreatment. In addition, Okabe et al (1989) suggested the possibility that additional pretreatment with rhG-CSF might render the neutropenia more serious compared with neutropenia without rhG-CSF pretreatment. However, the

© Cancer Research Campaign 1998

**Figure 6** Effects of pre- and post-treatment with rhG-CSF on neutrophil counts and progenitor cell counts in group A. As a pretreatment, rhG-CSF was administered from day −2 to −1. Each data point represents the mean and bars s.d. ($n$ = 6, *$P$ < 0.05 vs control group, **$P$ < 0.05 vs control group) respectively. Open symbols, control; closed symbols, group A

**Figure 7** Effects of pre- and post-treatment with rhG-CSF on neutrophil counts and progenitor cell counts in group B. As a pretreatment, rhG-CSF was administered from day −4 to −3. Each data point represents the mean and bars represent s.d. ($n$ = 6, *$P$ < 0.01 vs control group, **$P$ < 0.05 vs control group) respectively. Open symbols, control; closed symbols, group B

expansion of progenitor cell populations, which was closely related to the peripheral neutrophil counts, was not evaluated. Moreover, the effects of specific timing of rhG-CSF pretreatment with respect to expansion of progenitor cell populations when chemotherapeutic agents have been administered has not been thoroughly investigated.

In the present study, the effects of altering the timing of rhG-CSF administration with respect to CPA treatment were examined. The post-treatment group was relevant to the conventional clinical situation in which rhG-CSF administration is initiated after chemotherapy (Gabrilove et al, 1988; Crawford et al, 1991; Sarosy et al, 1992). Although recovery of neutropenia was promoted in this group, an increase in the nadirs of neutrophil and total progenitor cell counts was not achieved compared with the control group, despite rhG-CSF administration. Neutrophil cell counts showed delayed changes after changes in progenitor cell populations in both the control and post-treatment groups, and this time lag suggests that neutrophil counts after CPA administration are dependent upon progenitor cell counts. Thus, the neutrophil cell count nadir after CPA administration seemed to be affected by the progenitor cell count nadir. With regard to the kinetics of progenitor cells, the suicide rate decreased to 0% in all groups and all of the progenitor cells disappeared completely after CPA administration. This suggests that only a part of progenitor cells outside the S-phase may survive when total progenitor cell counts have decreased to nadir after CPA administration. In addition, the subsequent remarkable increase in the suicide rate suggests that residual surviving progenitor cells that were outside the S-phase began active cell proliferation, leading to an increase in the total number of progenitor cells. This may occur because the recovery of total progenitor cells might be premature if the haemopoietic stem cells supported this increase in progenitor cell populations (Ikebuchi et al, 1988). As discussed previously, short-term rhG-CSF administration affected the kinetics of progenitor cell populations. Therefore, increases in the number of progenitor cells that are outside the S-phase as a result of rhG-CSF pretreatment before CPA administration, might lead to an increase in the number of residual surviving progenitor cells. This, in turn, could reduce the depression in total progenitor cell counts after CPA administration, and prevention of neutropenia could potentially be achieved.

To examine this possibility, we designed two protocols based on differences in the timing of rhG-CSF pretreatment in conjunction with CPA administration. In group A, in which CPA was administered when the number of progenitor cells outside the S-phase was at a minimum as a result of rhG-CSF pretreatment, the total number of progenitor cells, S-phase progenitor cells and neutrophil cells after CPA administration was more depressed than in the control group and the post-treatment group. This timing of rhG-CSF pretreatment seemed to render neutropenia much more serious than neutropenia in the absence of rhG-CSF treatment. In contrast, in group B, in which CPA was administered when the number of progenitor cells outside the S-phase had reached a peak, the nadir of the progenitor cell population was increased and recovery of the S-phase progenitor cell was

*© Cancer Research Campaign 1998*

promoted. In this group, neutrophil recovery was more accelerated than in the other groups and neutrophil recovery seemed to be supported by the increase in actively proliferating progenitor cells. These results suggest that the nadirs of neutrophil and progenitor cell counts after CPA administration are closely related to the number of progenitor cells outside the S-phase when CPA is administered, and that the effects of pretreatment with rhG-CSF differ in accordance with the timing of administration relative to CPA treatment.

Although these data were based on an experimental model, the possibility of protection against neutropenia by rhG-CSF pretreatment before CPA administration was observed. However, this depression did not appear to be due to neutrophil exhaustion before CPA administration (Morstyn et al, 1989). Rather, it appeared to be due to the decrease and delayed recovery of progenitor cell populations after CPA administration, which was caused by a decrease in the number of progenitor cells outside the S-phase. In contrast, the severity of neutropenia and the decrease in progenitor cell populations after CPA administration could be reduced by increasing the number of progenitor cells outside the S-phase when CPA was administered. This observation raises the possibility that more effective timing of rhG-CSF treatment, other than post-treatment, to reduce the severity of neutropenia after chemotherapy is possible. These observations also suggest that the kinetics of progenitor cell populations when chemotherapeutic agents are administered seems to play an important role in neutropenia after chemotherapy, and that not only peripheral neutrophil cell and total progenitor cell counts but also progenitor cell kinetics should be taken into consideration when administering rhG-CSF treatment against the effects of chemotherapy.

### REFERENCES

Bruning JL and Kintz BL (1987) *Computational Handbook of Statistics*, 3rd edn. pp. 275–278. Scott, Foresman: London

Crawford J, Ozer H, Stoller R, Johnson D, Lyman G, Tabbara I, Kris M, Grous J, Picozzi V, Rausch G, Smith R, Grandishar W, Yahanda A, Vincent M, Stewart M and Glapsy J (1991) Reduction by granulocyte colony-stimulating factor of fever and neutropenia induced by chemotherapy in patients with small-cell lung cancer. *N Engl J Med* **325**: 164–170

Gabrilove JL, Jakubowski A, Scher H, Sternberg C, Wong G, Grous J, Yagoda A, Fain K, Moore M, Clarkson B, Oettgen HF, Alton K, Welte K, and Souza LM (1988) Effect of granulocyte colony-stimulating factor on neutropenia and associated morbidity due to chemotherapy for transitional-cell carcinoma of the urothelium. *N Engl J Med* **318**: 1414–1422

Ikebuchi K, Clark SC, Ihle JN, Souza LM and Ogawa M (1988) Granulocyte colony-stimulating factor enhances interleukin 3-dependent proliferation of multipotential hemopoietic progenitors. *Proc Natl Acad Sci USA* **85**: 3445–3449

Morstyn G, Campbell L, Leishke G, Layton JE, Maher D, O'Connor M, Green M, Sheridan W, Vincent M, Alton K, Souza L, McGrath K and Fox RM (1989) Treatment of chemotherapy-induced neutropenia by subcutaneously administered granulocyte colony-stimulating factor with optimization of dose and duration of therapy. *J Clin Oncol* **17**: 1554–1562

Okabe T (1989) Application of human granulocyte colony-stimulating factor to cancer chemo- and radio-therapy. *Jpn J Inflammation* **9**: 231–239

Okabe M, Asano M, Kuga T, Komatsu Y, Yamasaki M, Yokoo Y, Itoh S, Morimoto M and Oka T (1990) In vitro and in vivo hematopoietic effect of mutant human granulocyte colony-stimulating factor. *Blood* **75**: 1778–1793

Sarosy G, Kohn E, Stone DA, Rothenberg M, Jacob J, Adamo DO, Ognibene FP, Cunnion RE and Reed E (1992) Phase I study of Taxol and granulocyte colony-stimulating factor in patients with refractory ovarian cancer. *J Clin Oncol* **10**: 1165–1170

Takaue Y, Kawano Y, Reading CL, Watanabe T, Abe T, Ninomiya T, Shimizu E, Ogura T, Kuroda Y, Yokobayashi A, Nakahata T, Asano S and Ventura G (1990) Effects of human G-CSF, GM-CSF, IL-3, and IL-1α on the growth of purified human peripheral blood progenitors. *Blood* **76**: 330–335

Tanaka H, Ishikawa R, Ishikawa M, Matsui S and Asano K (1991) Pharmacokinetics of recombinant human granulocyte colony-stimulating factor conjugated to polyethylene glycol in rats. *Cancer Res* **51**: 3710–3714

© Cancer Research Campaign 1998


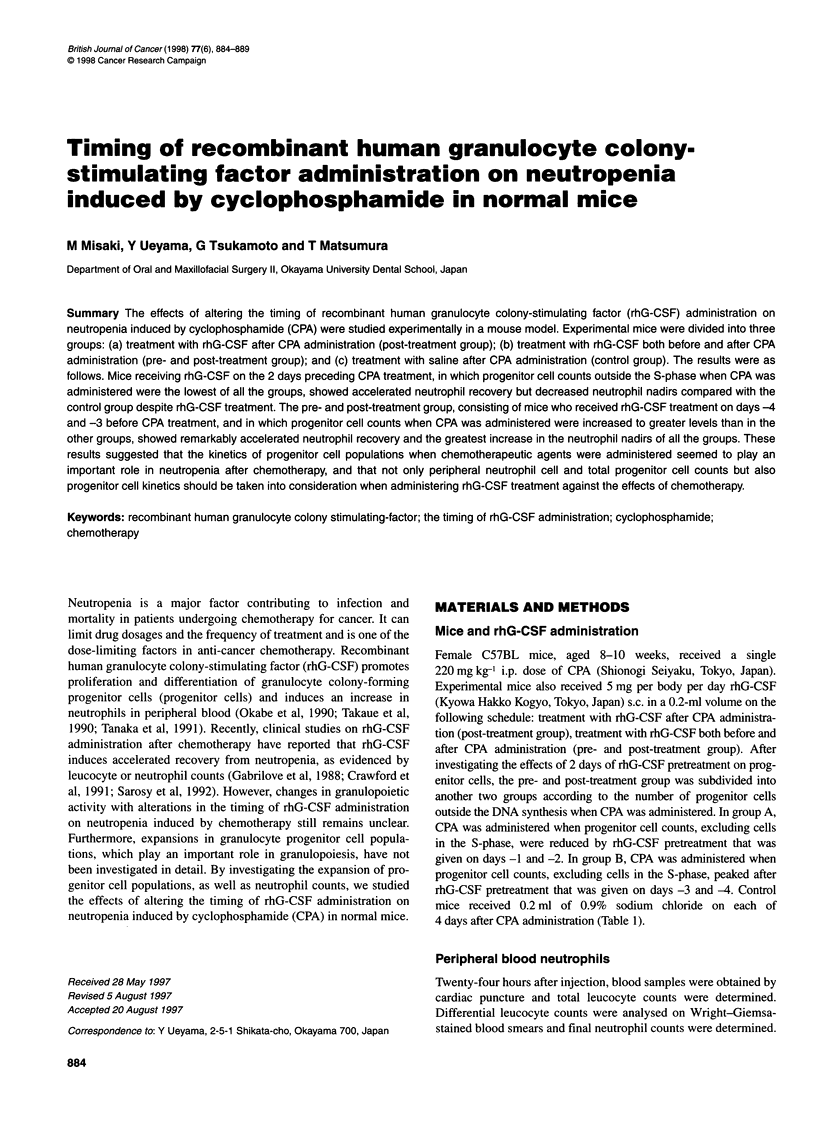

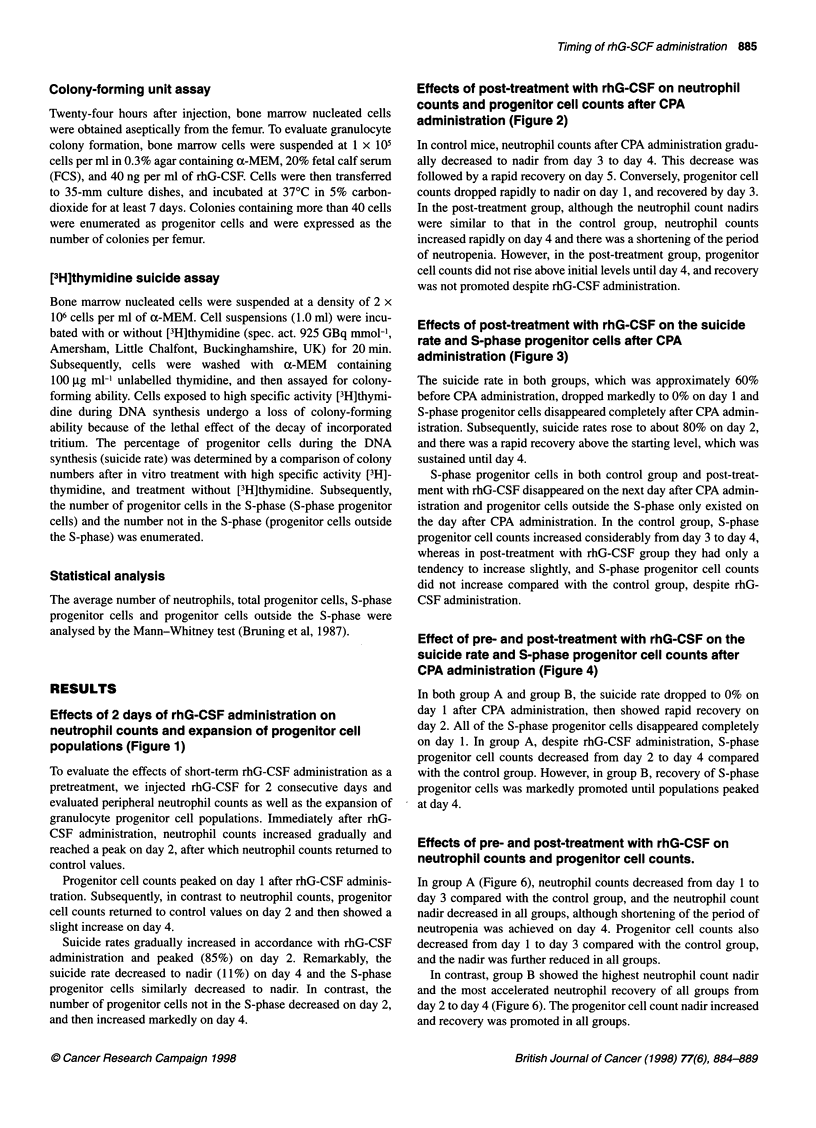

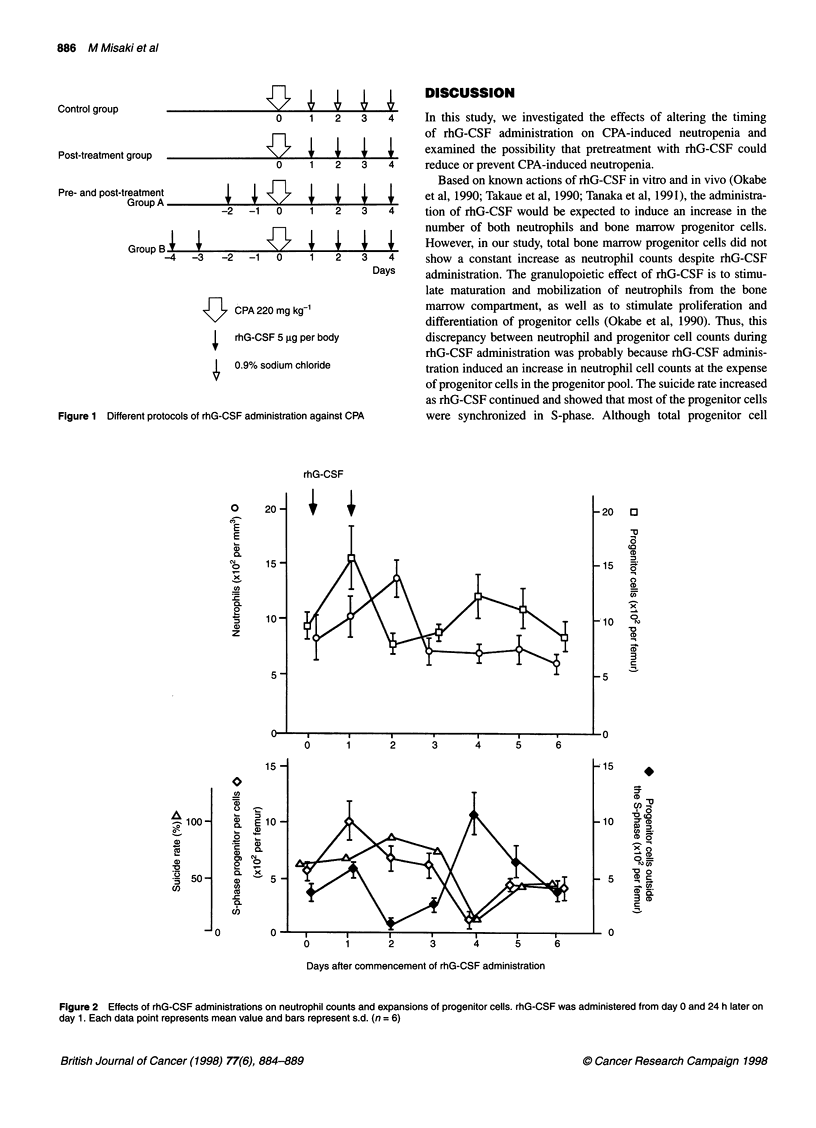

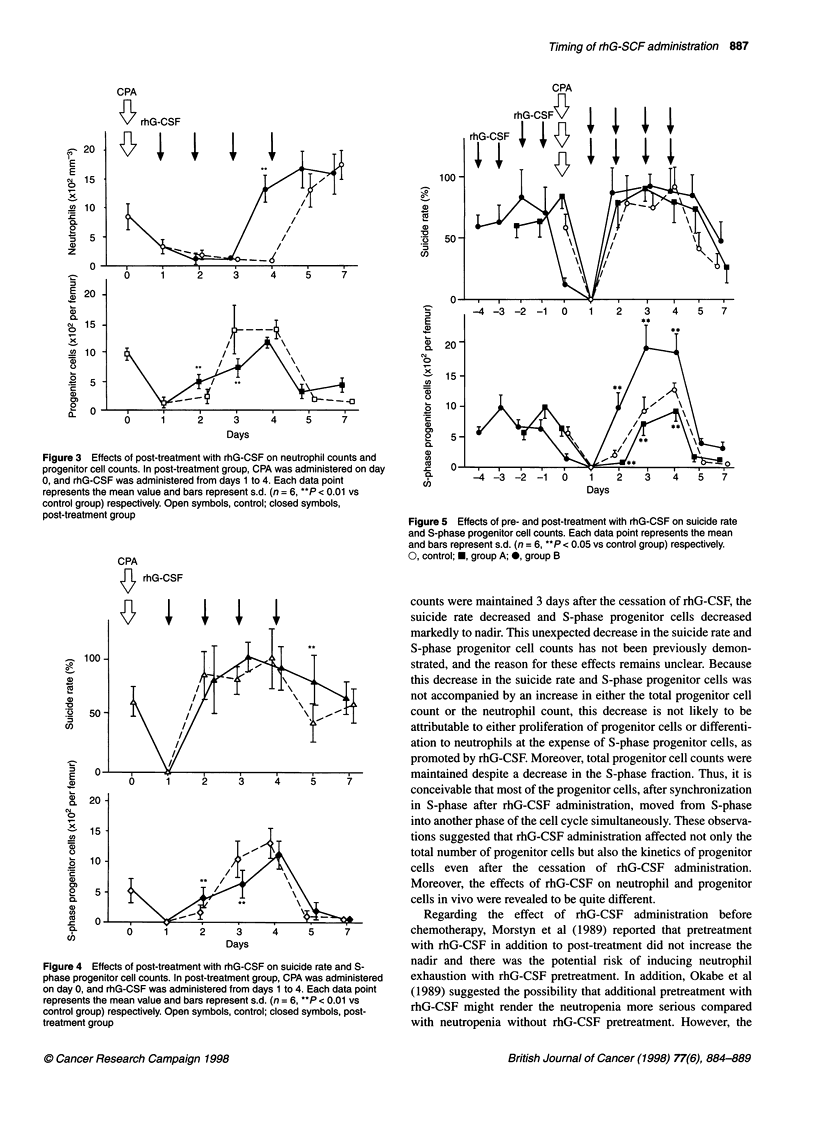

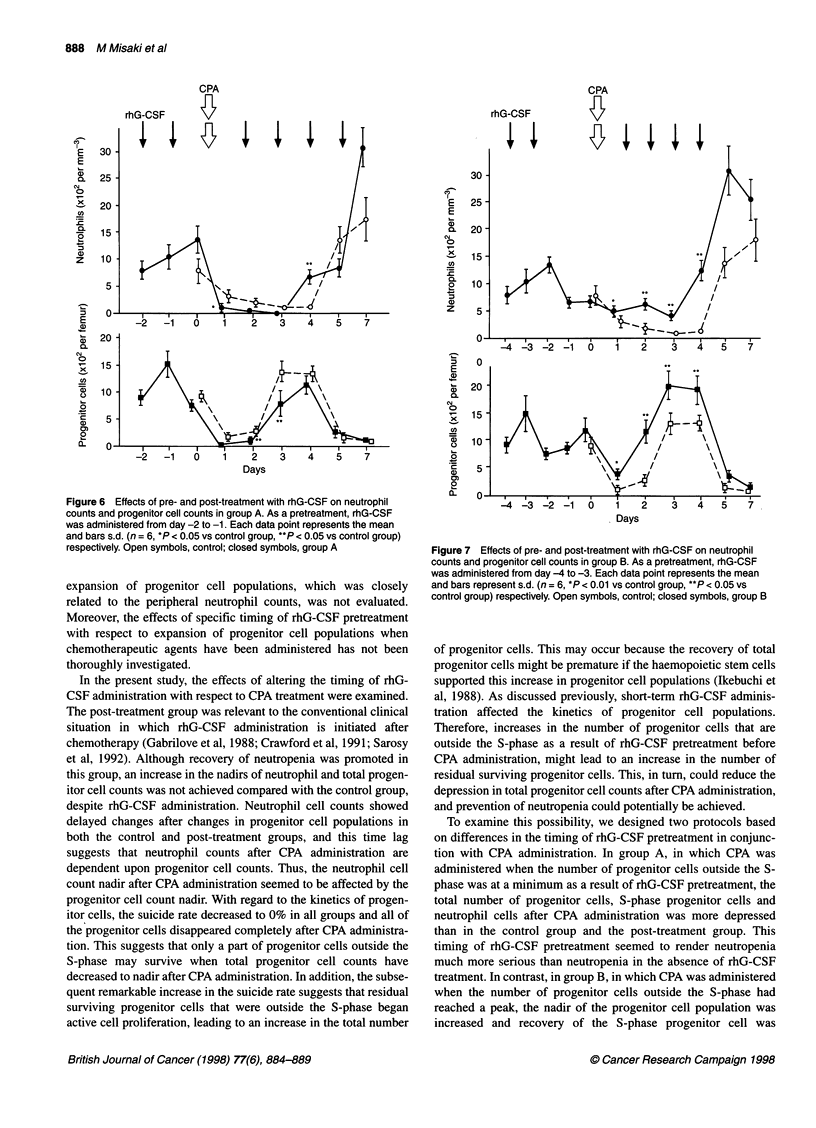

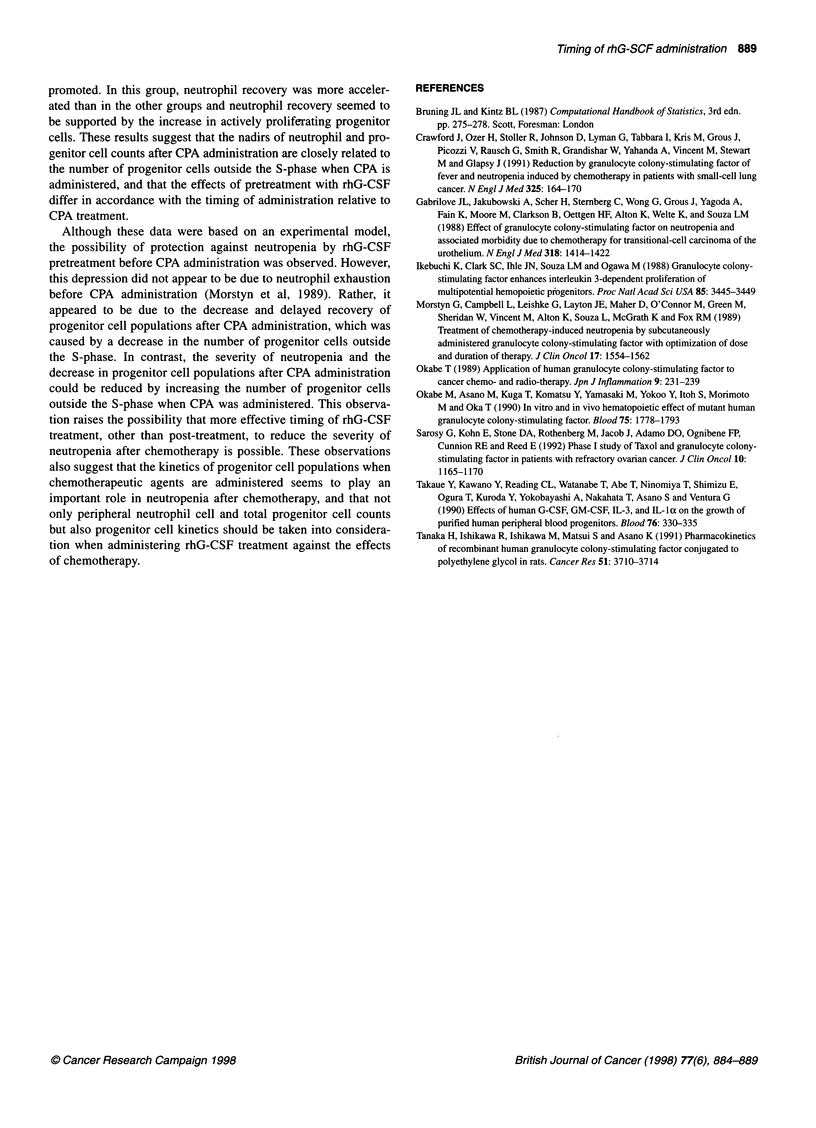


## References

[OCR_00875] Crawford J., Ozer H., Stoller R., Johnson D., Lyman G., Tabbara I., Kris M., Grous J., Picozzi V., Rausch G. (1991). Reduction by granulocyte colony-stimulating factor of fever and neutropenia induced by chemotherapy in patients with small-cell lung cancer.. N Engl J Med.

[OCR_00883] Gabrilove J. L., Jakubowski A., Scher H., Sternberg C., Wong G., Grous J., Yagoda A., Fain K., Moore M. A., Clarkson B. (1988). Effect of granulocyte colony-stimulating factor on neutropenia and associated morbidity due to chemotherapy for transitional-cell carcinoma of the urothelium.. N Engl J Med.

[OCR_00891] Ikebuchi K., Clark S. C., Ihle J. N., Souza L. M., Ogawa M. (1988). Granulocyte colony-stimulating factor enhances interleukin 3-dependent proliferation of multipotential hemopoietic progenitors.. Proc Natl Acad Sci U S A.

[OCR_00896] Morstyn G., Campbell L., Lieschke G., Layton J. E., Maher D., O'Connor M., Green M., Sheridan W., Vincent M., Alton K. (1989). Treatment of chemotherapy-induced neutropenia by subcutaneously administered granulocyte colony-stimulating factor with optimization of dose and duration of therapy.. J Clin Oncol.

[OCR_00908] Okabe M., Asano M., Kuga T., Komatsu Y., Yamasaki M., Yokoo Y., Itoh S., Morimoto M., Oka T. (1990). In vitro and in vivo hematopoietic effect of mutant human granulocyte colony-stimulating factor.. Blood.

[OCR_00913] Sarosy G., Kohn E., Stone D. A., Rothenberg M., Jacob J., Adamo D. O., Ognibene F. P., Cunnion R. E., Reed E. (1992). Phase I study of taxol and granulocyte colony-stimulating factor in patients with refractory ovarian cancer.. J Clin Oncol.

[OCR_00919] Takaue Y., Kawano Y., Reading C. L., Watanabe T., Abe T., Ninomiya T., Shimizu E., Ogura T., Kuroda Y., Yokobayashi A. (1990). Effects of recombinant human G-CSF, GM-CSF, IL-3, and IL-1 alpha on the growth of purified human peripheral blood progenitors.. Blood.

[OCR_00926] Tanaka H., Satake-Ishikawa R., Ishikawa M., Matsuki S., Asano K. (1991). Pharmacokinetics of recombinant human granulocyte colony-stimulating factor conjugated to polyethylene glycol in rats.. Cancer Res.

